# The Wzi outer membrane protein mediates assembly of a tight capsular polysaccharide layer on the *Acinetobacter baumannii* cell surface

**DOI:** 10.1038/s41598-021-01206-5

**Published:** 2021-11-05

**Authors:** Jacob Tickner, Sophia Hawas, Makrina Totsika, Johanna J. Kenyon

**Affiliations:** Centre of Immunology and Infection Control, School of Biomedical Sciences, Faculty of Health, Queensland University of Technology, Brisbane, Australia

**Keywords:** Bacterial genetics, Bacteria, Biofilms

## Abstract

Identification of novel therapeutic targets is required for developing alternate strategies to treat infections caused by the extensively drug-resistant bacterial pathogen, *Acinetobacter baumannii*. As capsular polysaccharide (CPS) is a prime virulence determinant required for evasion of host immune defenses, understanding the pathways for synthesis and assembly of this discrete cell-surface barrier is important. In this study, we assess cell-bound and cell-free CPS material from *A. baumannii* AB5075 wildtype and transposon library mutants and demonstrate that the Wzi outer membrane protein is required for the proper assembly of the CPS layer on the cell surface. Loss of Wzi resulted in an estimated 4.4-fold reduction in cell-associated CPS with a reciprocal increase in CPS material shed in the extracellular surrounds. Transmission electron microscopy revealed a disrupted CPS layer with sparse patches of CPS on the external face of the outer membrane when Wzi function was lost. However, this genotype did not have a significant effect on biofilm formation. Genetic analysis demonstrated that the *wzi* gene is ubiquitous in the species, though the nucleotide sequences were surprisingly diverse. Though divergence was not concomitant with variation at the CPS biosynthesis K locus, an association between *wzi* type and the first sugar of the CPS representing the base of the structure most likely to interact with Wzi was observed.

## Introduction

Carbapenem-resistant *Acinetobacter baumannii* is listed as a critical priority bacterial pathogen by the World Health Organization (WHO)^1^, identifying it as species for which innovative therapeutic strategies are urgently needed. Extensive and pan antibiotic-resistant *A. baumannii* infections are not only associated with poorer patient outcomes with significant morbidity and mortality but are also difficult to eradicate from clinical environments despite infection control procedures^2^. The success of the species as a serious global pathogen can be attributed to a highly plastic genome with significant mutation rates and frequent acquisition of genes that confer extensive antimicrobial resistance, increase virulence in the host, and/or enhance survival in unfavorable conditions over long periods^3,4^. These characteristics have favored the expansion of multi-drug resistant clonal lineages, including the two globally disseminated clones, global clone 1 (GC1) and global clone 2 (GC2), emphasizing a need to better understand mechanisms for virulence and survival in host and nosocomial contexts.

Bacterial protection from external antimicrobial threats is known to be significantly enhanced by the presence of a complex polysaccharide matrix, known as the capsular polysaccharide (CPS). CPS forms the external-most layer of the bacterial cell envelope and is comprised of high molecular weight polymers of repeating oligosaccharide units (K units)^5,6^. CPS is a primary virulence determinant and its presence is critical for protection against complement-mediated killing and opsonophagocytosis^7–10^, as well as for mediating resistance to desiccation and other antimicrobials^11–13^. Thus, removing or damaging the CPS barrier is a promising strategy to attenuate or re-sensitize the bacterium to specific antimicrobials.

Biosynthesis of the *A. baumannii* CPS follows a generalized Wzy-dependent pathway for K-unit construction, polymerization and export to the extracellular side of the gram-negative outer membrane^5^. The majority of genes responsible for CPS biosynthesis and export are arranged in a cluster located at the chromosomal K locus^5^. More than 128 distinct gene clusters have been identified at this location^14^, predicting extensive structural heterogeneity of the CPS between different isolates. This complicates vaccine and phage therapies that target this structure. However, despite the structural variation, the exact mechanism for attachment of all CPS structures to the cell surface remains unknown.

**Figure 1:**
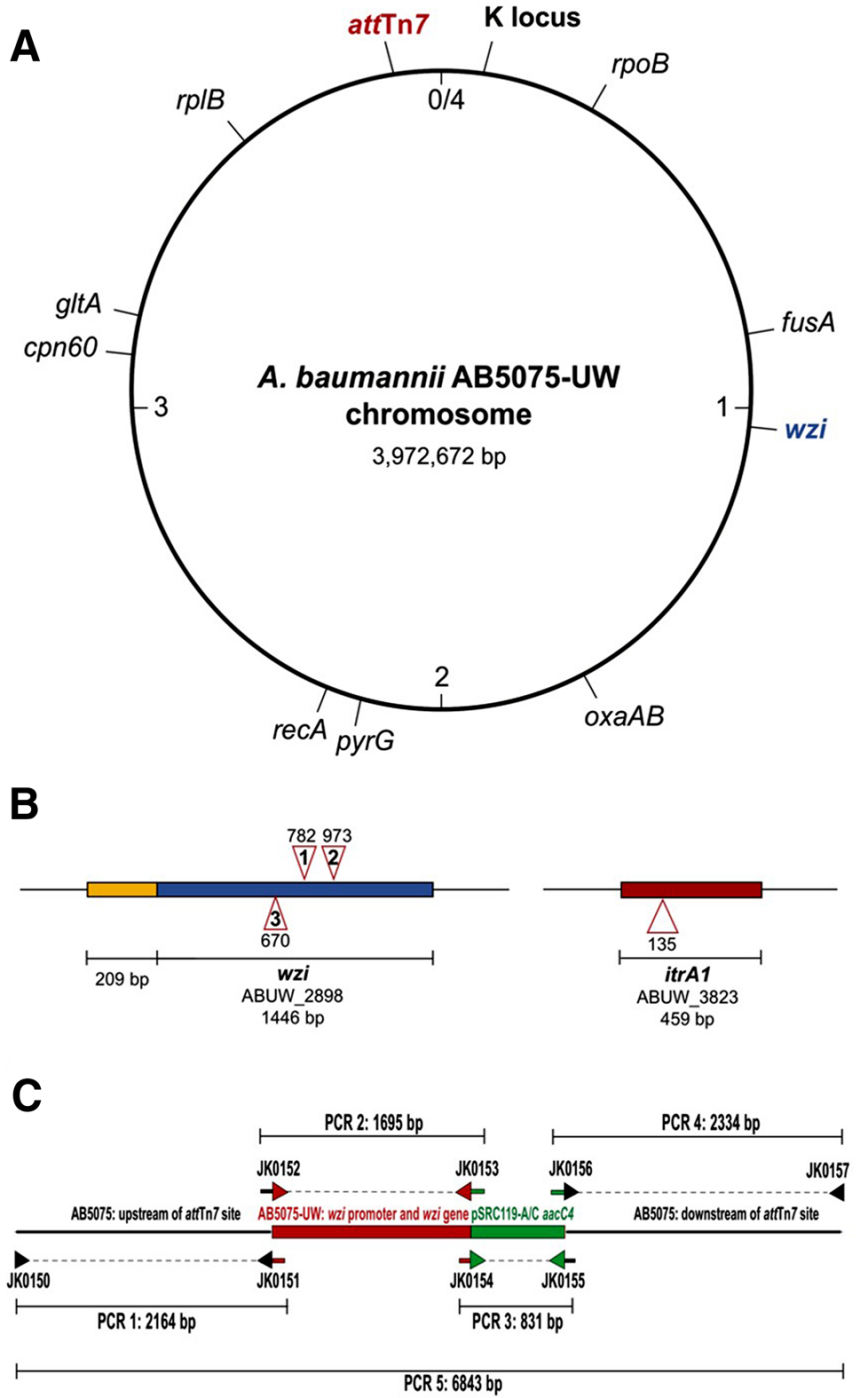
(**A**) Representation of the *A. baumannii* AB5075-UW chromosome showing location of *wzi* gene (blue) in relation to the K locus and *att*Tn*7* site (red). Institut Pasteur MLST alleles and the intrinsic *oxaAB* gene are also shown. (**B**) Genetic location of T26 insertion sites in *A. baumannii* AB5075-UW transposon library mutants. Genes, locus tags, and sequence lengths are indicated below the gene. Triangles represent insertion sites at listed base positions in gene sequence. Insertions in the forward strand shown above and insertions in reverse orientation shown below. The intergenic region that includes a predicted promoter sequence for *wzi* (blue) is shown upstream (yellow). (**C**) Composition of the chimeric gene cassette used to complement *wzi::T26* mutant strains. Size of amplicons are indicated along with the positions of oligonucleotide primers used for amplification. Oligonucleotide sequences are listed in Supplementary Table S5.

In *Escherichia coli* O9a:K30*,* the K30 CPS is synthesized via the same Wzy-dependent pathway, and assembly of this structure on the cell surface is known to be mediated by a Wzi outer membrane protein^15^. When Wzi function is abolished, a marked reduction in cell-associated CPS is observed with a corresponding increase in the amount of CPS found in the external surrounds^16^. Previously, a candidate *wzi* gene, whose product shares 48% amino acid sequence identity with Wzi from *E. coli* O9a:K30, was identified in the *A. baumannii* chromosome^5^, and it was proposed that association of *A. baumannii* CPS with the cell-surface may be controlled by the same machinery. This candidate gene is found at a different location away from the variable K locus in the *A. baumannii* chromosome (Fig. 1A).

In this study, we evaluate the role of the candidate Wzi protein in the association of CPS with the *A. baumannii* cell surface and assess the effect that CPS shedding has on biofilm formation. The diversity of *wzi* sequence between different isolates is also examined.

## Results

### Validation and complementation of *A. baumannii* wzi mutants

The AB5075-UW wildtype (GenBank accession number CP008706.1), three AB5075-UW mutants carrying T26 insertions in *wzi* (locus tag ABUW_2898)*,* and one AB5075-UW mutant with a T26 insertion in *itrA1* (locus tag ABUW_3822) representing a CPS-negative phenotype were acquired for analysis (see Table 1). Genomic material from each mutant strain was sequenced and the precise genetic context of the T26 insertion was confirmed within the correct gene for all mutants (Fig. 1B). Whole genome sequences were further investigated for other differences from the wildtype AB5075-UW reference genome, and only one single nucleotide polymorphism (SNP) was identified in each genome assembly. The SNP was located away from any gene known to be involved in CPS biosynthesis^5^, indicating that T26 mutants shared an isogenic background with the AB5075 wildtype.

**Table 1:** Bacterial strains and plasmids used in this study.

Name	Strain and characteristics	References
WT	*Acinetobacter baumannii* AB5075-UW	^27^
∆*itrA1*::T26	*Acinetobacter baumannii* AB5075 tnab1_kr140805p02q177, T26 insertion in *itr*A1 (ABUW_3823)	^27^
∆*wzi*::T26 - 1	*Acinetobacter baumannii* AB5075 tnab1_kr130916p01q164, T26 insertion in *wzi* (ABUW_2898)	^27^
∆*wzi*::T26 - 2	*Acinetobacter baumannii* AB5075 tnab1_kr121204p02q167, T26 insertion in *wzi* (ABUW_2898)	^27^
∆*wzi*::T26 - 3	*Acinetobacter baumannii* AB5075 tnab1_kr121203p06q189, T26 insertion in *wzi* (ABUW_2898)	^27^
∆*wzi*::T26 attTn7-*wzi* - 1	*Acinetobacter baumannii* AB5075 tnab1_kr130916p01q164, T26 insertion in *wzi* (ABUW_2898) and *wzi-aacC4* insertion at *att*Tn7 site	This study
∆*wzi*::T26 attTn7-*wzi* - 2	*Acinetobacter baumannii* AB5075 tnab1_kr121204p02q167, T26 insertion in *wzi* (ABUW_2898) and *wzi-aacC4* insertion at *att*Tn7 site	This study
∆*wzi*::T26 attTn7-*wzi* - 3	*Acinetobacter baumannii* AB5075 tnab1_kr121203p06q189, T26 insertion in *wzi* (ABUW_2898) and *wzi-aacC4* insertion at *att*Tn7 site	This study
pSRC119-A/C	*Salmonella enterica* pSRC119-A/C plasmid vector carrying an *aacC4* apramycin resistance gene. Sequence available in GenBank accession number KM670336.1	^41^

Each *wzi::T26* mutant was complemented by the insertion of a chimeric gene cassette into the stable *att*Tn*7* site in the AB5075-UW chromosome in order to restore Wzi function to natural levels with single copy number expression. The cassette (Fig. 1C) included a short sequence of the intergenic space upstream of *wzi* in the AB5075-UW genome, a complete copy of the wildtype *wzi* gene, and an *aacC4* gene conferring apramycin resistance for selection. The design of the cassette to include the intergenic sequence upstream of *wzi* provided the putative native promoter to enable natural control of its expression under the tested growth conditions.

### Wzi is required for retention of CPS on the cell surface

**Figure 2:**
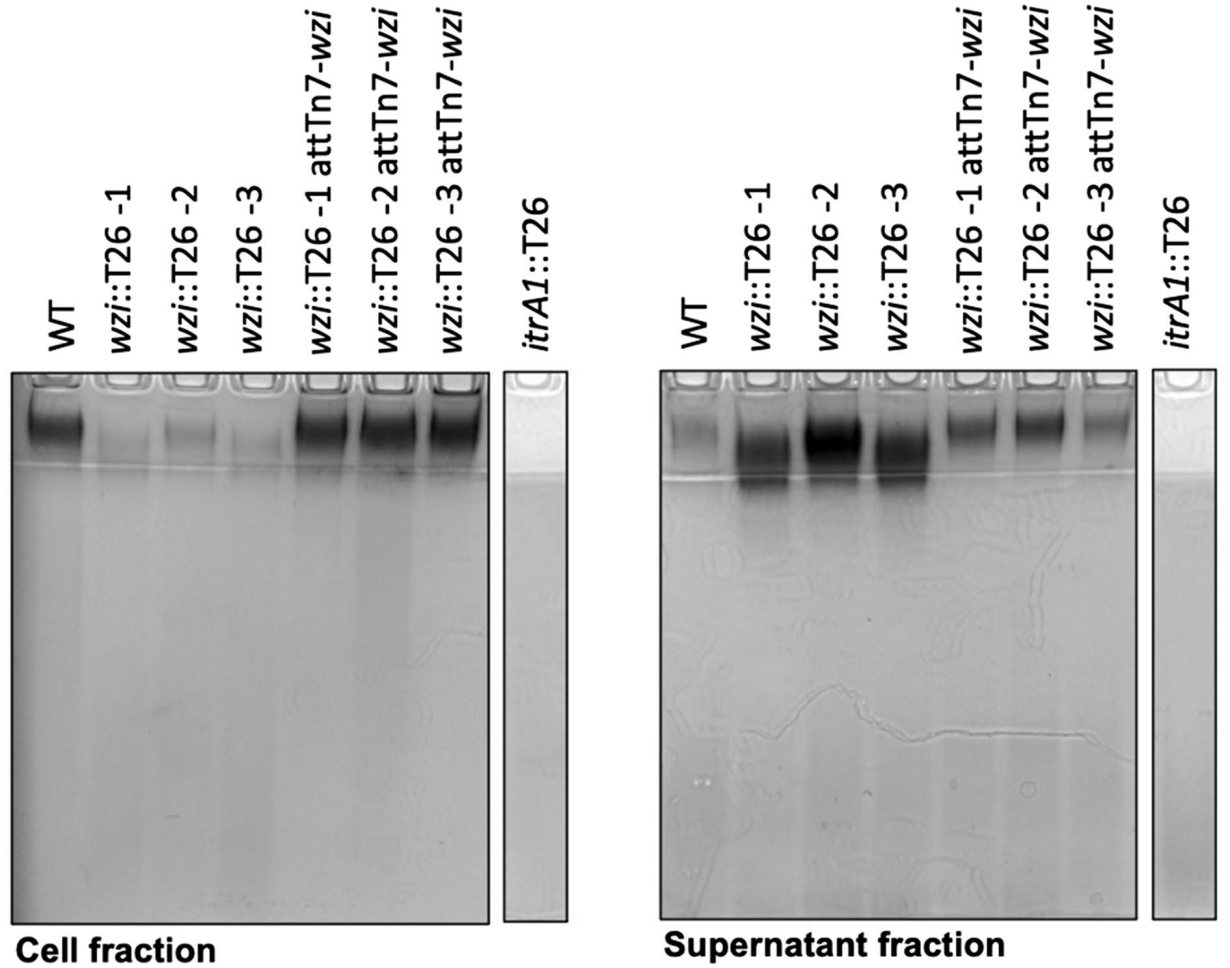
CPS phenotypes of *A. baumannii* wildtype, *wzi* and *itrA1* mutants. Purified capsular polysaccharide from *A. baumannii* cells and supernatants. Samples were visualized by SDS-PAGE and stained with Alcian blue. Cell fraction represents the proportion of CPS attached to the cell surface, while polysaccharide in the supernatant represents CPS shed from the cell surface. Strain names are shown above each well. Original uncropped gel images are provided in Fig. S3.

The role of Wzi in the association of CPS with the cell surface was first investigated by comparing CPS material purified from harvested cells (cell-associated CPS) with that of the supernatant (CPS released from the cell surface) from the same culture. SDS-PAGE analysis of extracts from both fractions (Fig. 2) showed the presence of high molecular weight CPS molecules, located close to the interface between stacking and separating gels consistent with previous studies^10,17,18^. The AB5075-UW wildtype displays an abundance of cell-associated CPS with an estimated 19.5% of the total CPS material (combined relative density of cell and supernatant fractions) found in the supernatant fraction. This suggests that a low level of CPS shedding naturally occurs in this strain. CPS material was not observed for either cell or supernatant fractions of the *itrA1::T26* mutant consistent with a CPS-negative phenotype, confirming the role of ItrA1 in the synthesis of the AB5075 CPS.

An estimated 4.4 fold reduction in cell-associated CPS is observed for all three *wzi::T26* mutants in comparison to the wildtype, with 80–85% of total CPS material observed in the supernatant fractions. However, 15–20% of the total CPS produced remains present in the cell fraction of each strain, suggesting that loss of Wzi reduces but does not eliminate CPS on the cell surface. Each complemented *wzi::T26 att*Tn*7-wzi* strain displayed CPS phenotypes equivalent to that of the wildtype indicating successful restoration of Wzi function. These results suggest that Wzi plays an important role in retaining CPS molecules on the cell surface.

### Wzi mediates assembly of a dense CPS layer on the cell surface

**Figure 3:**
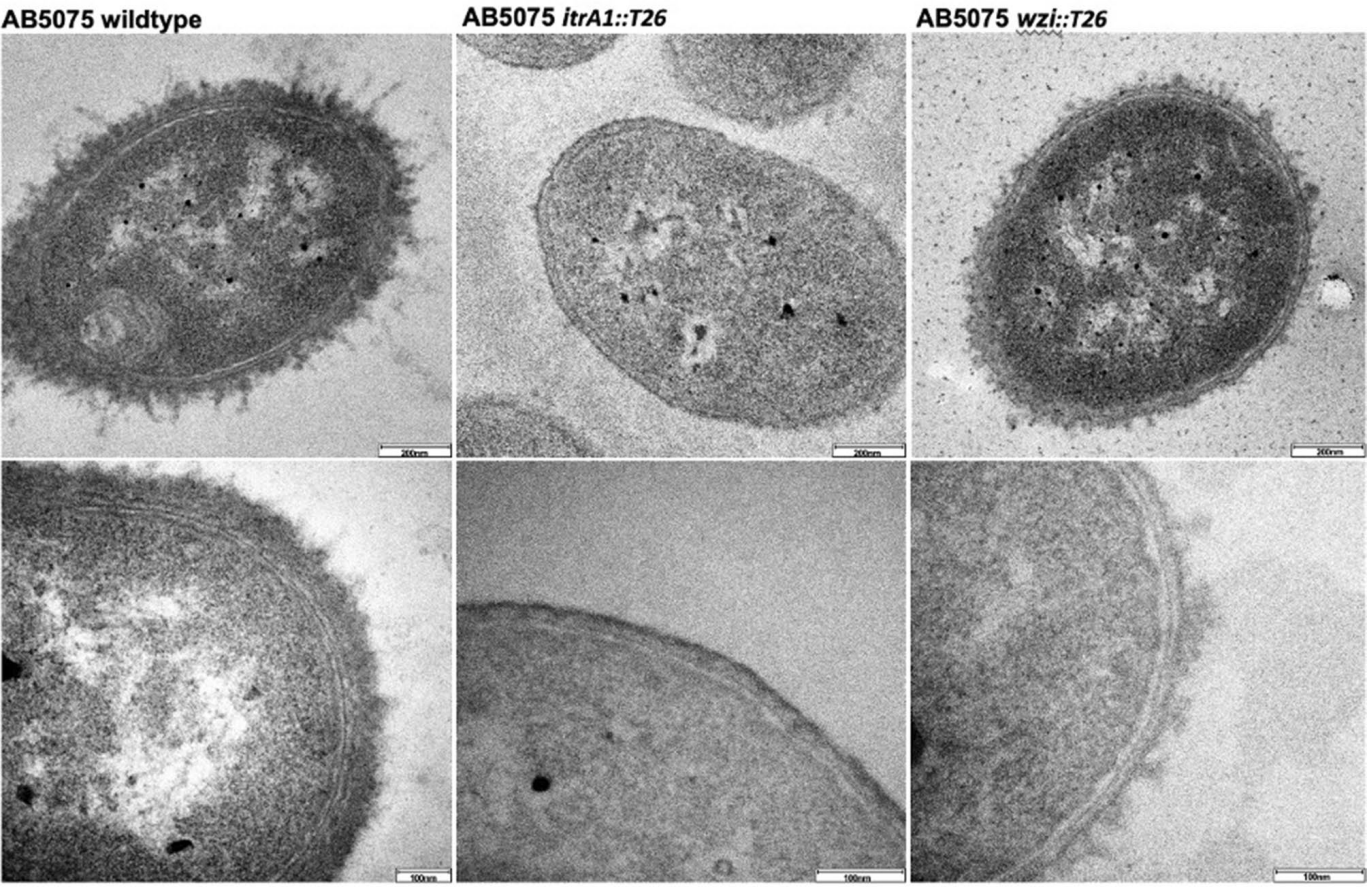
Transmission electron microscopy images of *A. baumannii* AB5075 wildtype, and representative *wzi* and *itrA1* mutants. Cells stained with ruthenium red to visualize CPS. Top row shows an entire representative cell from each strain with a 200 nm scale. Bottom row is the magnified cell surface of each strain with a 100 nm scale.

To directly visualize the effect of Wzi loss on the integrity of the CPS layer on the intact cell surface, cell sections of the wildtype, *itrA1::T26* mutant and *wzi::T26-1* mutant were examined using transmission electron microscopy (TEM). Consistent with SDS-PAGE analysis, the AB5075-UW wildtype (Fig. 3) displays a dense CPS layer with a mean thickness of 85.7 nm comparable to CPS-positive phenotypes of *A. baumannii* isolates examined in other studies ^18–20^. A CPS-negative phenotype is observed for the *itrA1::T26* mutant as expected, whereas the cell surface of the *wzi::T26-1* mutant shows a disrupted CPS layer with sparse patches of CPS on the external face of the outer membrane. This indicates that Wzi is involved in the assembly of a tight CPS layer on the cell surface.

### CPS presence rather than retention on the cell surface influences biofilm formation

**Figure 4:**
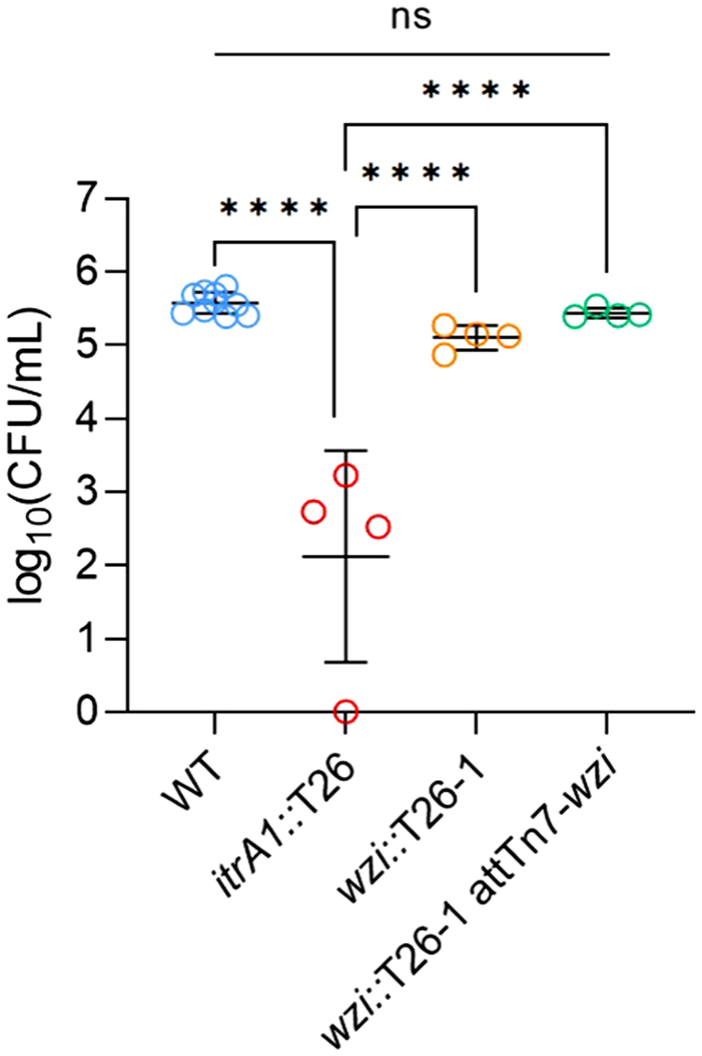
Biofilm formation by *A. baumannii* wildtype and representative *wzi* and *itrA1* mutants. Cells were recovered from biofilms grown on MBEC pegs for 24 h at 37 °C in LB. Viable CFU mL^−1^ were enumerated by serial dilution and plating on LB agar. Dot plots show data from at least 2 biological repeats each with 2 technical replicates. Horizontal lines show group means and standard deviation. The data were analysed by an ordinary one-way ANOVA with Tukey’s multiple comparisons post hoc test. ****P < 0.0001, ns, not significant.

In previous studies, deletion of CPS biosynthesis genes in *A. baumannii* strains has been shown to reduce biofilm formation or significantly alter biofilm morphology^8,20^. Therefore, we conducted an examination of the ability of the *wzi::T26-1* mutant to form a biofilm. Following growth for 24 h in the MBEC device (formerly the Calgary Biofilm Device), the AB5075-UW wildtype formed cell-dense biofilm communities (Fig. 4), which is consistent with previous reports for this strain^21^. In comparison, the *itrA1::T26* mutant showed a significant decrease in biofilm cell density as expected for a CPS-negative strain (*P* < 0.0001, ANOVA). However, the *wzi::T26-1* and *wzi*::*T26*-1 *att*Tn*7*-*wzi* strains each formed biofilms of equivalent cell density to that of the wildtype, indicating that extracellular presence of CPS material rather than its association with the cell surface is important for the formation of biofilm.

### The *wzi* gene exhibits unexpected sequence diversity

The requirement of Wzi to assemble a tight CPS layer on the cell surface raises the possibility of a specific interaction between Wzi and the CPS structure. Considering the heterogeneous nature of the CPS, diversity in Wzi was also examined. A total of 9342 *A. baumannii* genome assemblies from the NCBI non-redundant and WGS databases were acquired for analysis. Each genome assembly was found to include a *wzi* gene sequence sharing 100% coverage and > 82% nucleotide sequence identity with AB5075-UW *wzi* (Supplementary Table S1). Eight assemblies included a second *wzi* gene at the K locus, referred to as *wzi*_*KL*_, which was found together with an *itrA4* gene coding for a D-galactose (D-Gal) 1-phosphate initiating transferase described in a recent study^22^.

To assess *wzi* diversity in the species, a selection of 109 *wzi* nucleotide sequences, including five *wzi*_*KL*_ sequences, were extracted for further analysis. Sequences selected represented a diverse pool of *A. baumannii* isolates that belong to a range of clonal lineages and carry different CPS biosynthesis genes at the K locus (details in Supplementary Table S2). In addition, as the first sugar of the CPS is the base of the structure proximal to the cell surface that is most likely to interact with Wzi, the first sugar of each CPS type was also predicted. This was achieved via the identification of the Itr initiating transferase encoded by the CPS biosynthesis gene cluster in each genome (Supplementary Table S2). In *A. baumannii*, the linkage of the first sugar to the inner membrane lipid carrier to begin CPS synthesis is catalysed by one of six Itr initiating transferase enzymes, for which the sugar substrates have been experimentally confirmed or deduced for > 65 *A. baumannii* CPS structures determined to date. Itr enzymes and their associated sugar substrates are listed in Table 2.

**Table 2:** Initiating transferase sugar specificities.

Itr name	First sugar	References
ItrA1	D-Qui*p*NAc4NR^a^	^42,43^
ItrA2	D-Gal*p*NAc	^8,43–45^
ItrA3	D-Glc*p*NAc	^43,46^
ItrA4	D-Gal*p*	^22^
ItrB1	D-Qui*p*NAc	^47^
ItrB2	–^b^	–
ItrB3	D-Fuc*p*NAc	^48^

^a^R is either Acetyl (Ac) or (S)-3-hydroxybutanoyl.

^b^ItrB2 is redundant.

**Figure 5:**
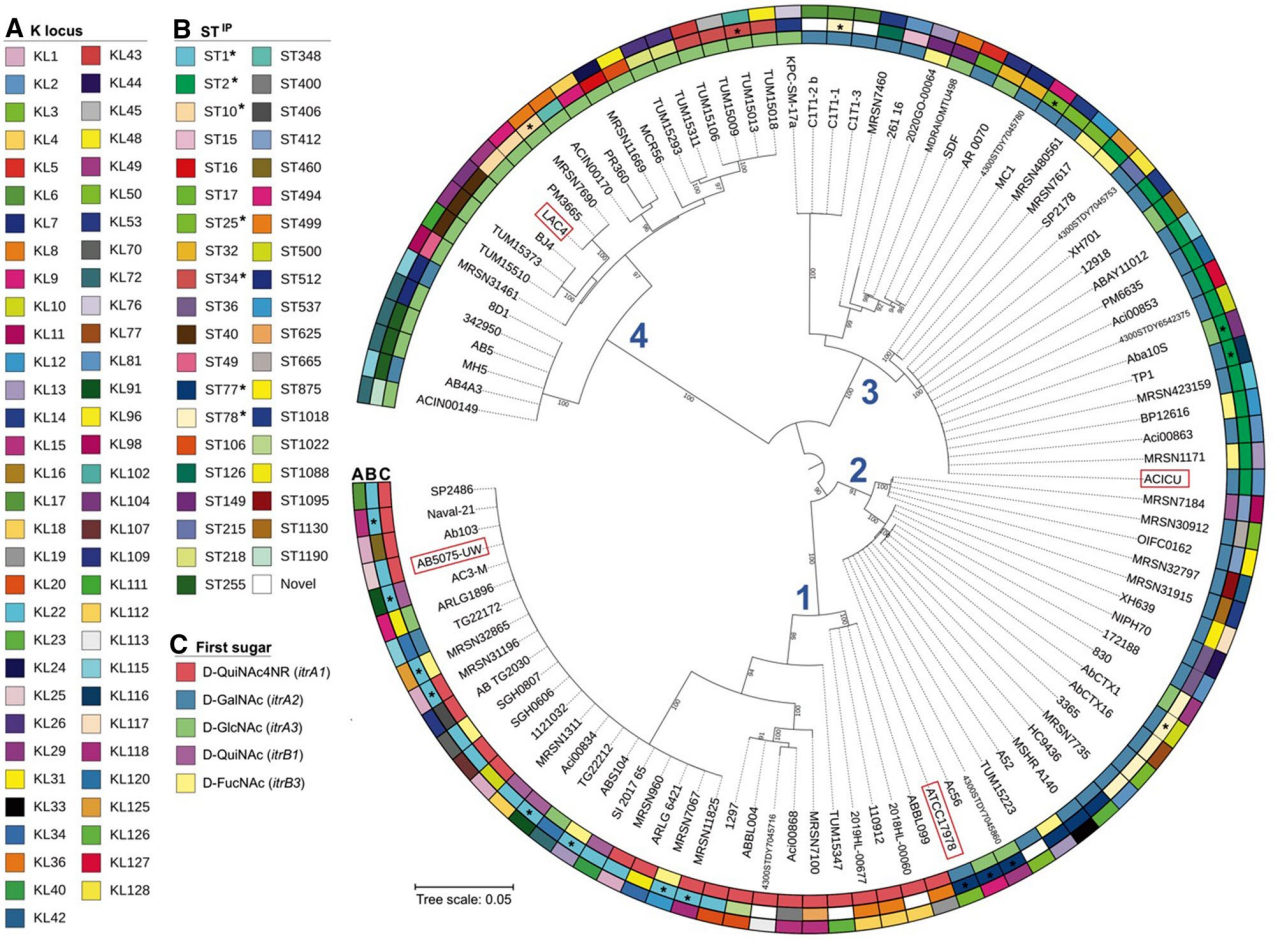
Phylogeny of *wzi* sequences in a diverse collection of *A. baumannii* genomes. Strain names are shown. Surrounding rings indicate the K locus type (ring A), Institut Pasteur sequence type (ST^IP^, ring B), and predicted or known first sugar of the CPS unit produced by the respective KL genes (ring C), and colour scheme is shown on the left. Blue numbers (1–4) indicate major phylogenetic clades. * denotes single locus variants (SLV) of sequence types.

A maximum likelihood tree (Supplementary Fig. S1) of the 109 *wzi* nucleotide sequences revealed five distinct phylogenetic clades, one of which occurred as a separate lineage and included all five *wzi*_*KL*_ sequences. Sequences from the *wzi*_*KL*_ clade share 64–67% nucleotide sequence identity with *wzi* sequences from the other four clades (Supplementary Table S3), suggesting an import of the *wzi*_*KL*_ sequence from outside the species as proposed previously^22^. Therefore, a second maximum likelihood tree was constructed with only *wzi* sequences located at the chromosomal *wzi* locus. Four major phylogenetic clades were again observed defining four *wzi* sequence types (Fig. 5, numbered in blue). Sequences belonging to the same clade/type share > 92% nucleotide sequence identity (Supplementary Table S3) indicating a conserved relationship.

The AB5075-UW *wzi* sequence was found to belong to a homogenous lineage within clade 1, which includes mostly isolates belonging to GC1 (ST1 and single locus variants, SLVs, of ST1). Though there is a diverse array of STs (ring B in Fig. 5) represented in each clade, GC2 (ST2 and SLVs) isolates group together in Clade 3 along with isolates belonging to ST25, another important multi-drug resistant clonal lineage^23^. A total of 64 KL types are also represented in the phylogeny (ring A, Fig. 5), and interestingly, there is no observed association between the clade and KL type, suggesting that the overall composition and/or topology of the CPS structure is irrespective of the *wzi* sequence.

A larger proportion of isolates within the same *wzi* clade encoded the same *itr* initiating transferase gene (shown in ring C, Fig. 5). For example, most isolates with *wzi* type 1 (clade 1) carry an *itrA1* gene predicting *N*-acetylbacillosamine (D-QuiNAc4NR) as the first sugar of the CPS structure. In comparison, the *itrA2* gene, which predicts a *N*-acetylgalactosamine (D-GalNAc) first sugar, is observed more frequently with *wzi t*ype 2 (clade 2) and *wzi* type 3 (clade 3), while *itrA3* predicting a *N*-acetylglucosamine (D-GlcNAc) first sugar is more often found in isolates with *wzi* type 4 (clade 4). This observed trend suggests that *wzi* sequence types may have an association with the type of proximal sugar at the base of the CPS structure.

### Co-occurrence of *wzi* and *itr* gene types

**Figure 6:**
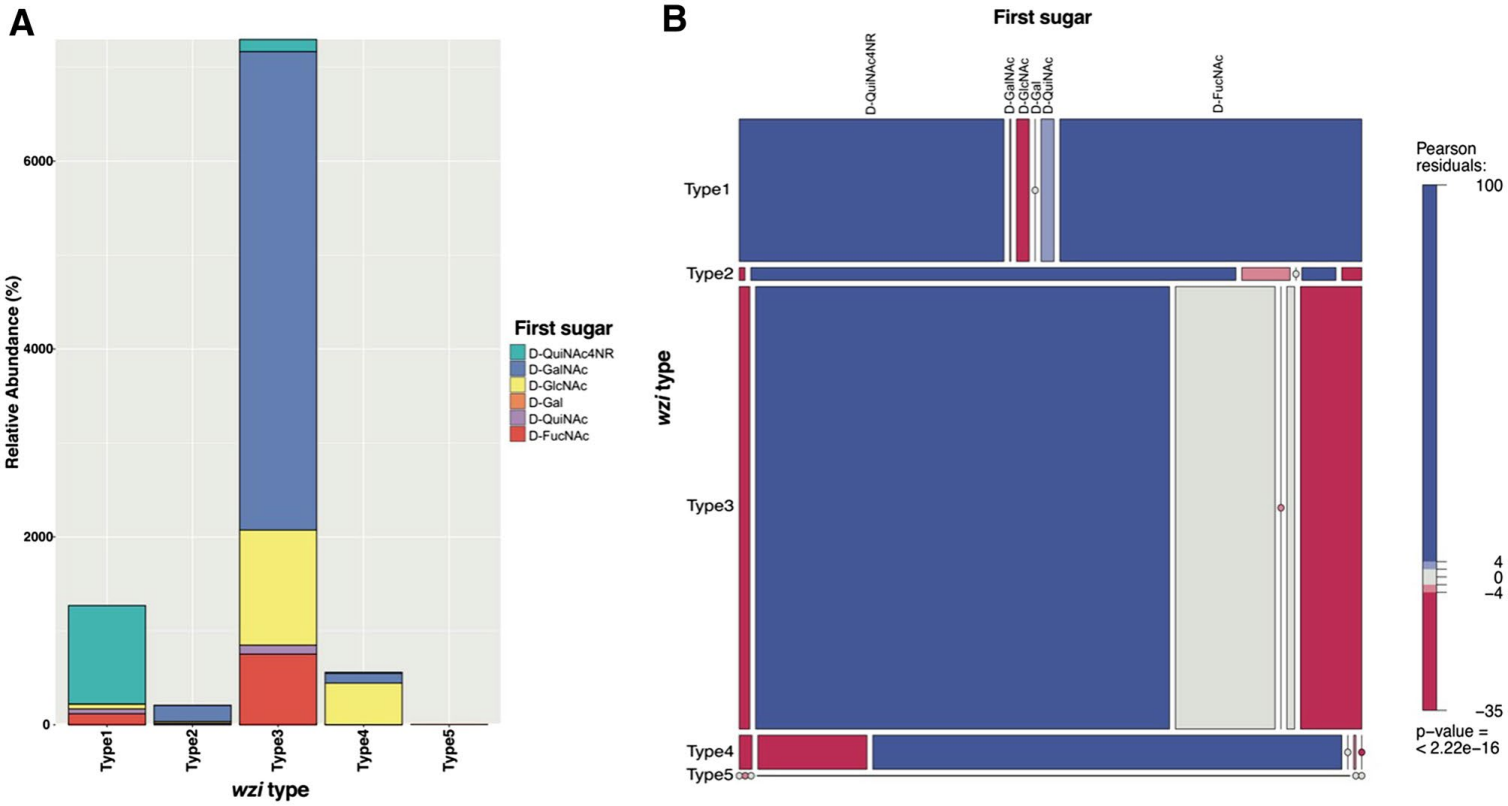
Association between *wzi* and *itr* types. (**A**) Stacked bar plot showing the relative abundance of *itr* types found in *A. baumannii* genomes grouped by *wzi* type. Size of the box denotes the relative number of genomes with the indicated *itr* type (coloured according to scheme shown on the right). (**B**) Mosaic plot demonstrating the association between observed frequencies of *wzi* and *itr* types. The width and height of each tile indicates the percentage of genomes with the two categorical variables shown on the x and y axes, respectively. Colours represent standardized residuals of a chi-square test with scale shown on the right. Blue denotes occurrences of *wzi* and *itr* combinations that are over-represented, whereas red are under-represented occurrences. Circles are a count of zero.

To more widely test *wzi*-*itr* gene associations, the relative frequency of *wzi* types co-occurring with specific *itr* genes was examined in 9342 *A. baumannii* genome assemblies (Supplementary Table S1). The number of genomes encoding each *wzi* type was examined relative to the number of genomes encoding each *itr* gene (Supplementary Table S4) and plotted in Fig. 6A. With the exception of *wzi*_*KL*_ type 5, which only co-occurred with *itrA4* (see above), almost all *itr* genes can be found in genomes carrying each *wzi* type. However, the majority of genomes (n = 7295) possess *wzi* type 3 (clade 3), of which 5092 (~ 70%) also carry *itrA2* (Fig. 6A). The second most common was *wzi* type 1 (n = 1270) with 1047 genomes (82%) encoding *itrA1* and 119 (9%) having *itrB3*. Type 2 and type 4 *wzi* were found more frequently in genomes with *itrA2* and *itrA3*, respectively.

The frequency of *wzi* types co-occurring with *itr* genes was further visualized by a mosaic plot (Fig. 6B), which displays the results of a chi-squared test for independence with colours indicating deviation from the expected frequency (residual). The plot in Fig. 6B shows significant positive residuals for seven different combinations of *wzi* and *itr* types (blue tiles), with the *wzi* type 3/*itrA2* combination displayed as the largest observed group (shown by the largest tile size). The observed association between *wzi* and *itr* sequences suggests that Wzi types may have specificity for the proximal sugar of the CPS substrate should interaction between the two molecules exist.

### The predicted tertiary structure of Wzi

A single isolate from each *wzi* clade was chosen as a representative of each Wzi type (boxed in red in Fig. 5), and a percentage identity matrix of the translated Wzi sequences showed that the amino acid sequence identities of types 1 to 4 ranged between 88.12% and 93.75% (Table 3). A multiple sequence alignment (Supplementary Fig. S2) indicated that sequence differences in the four Wzi types lie predominately in the central portion (amino acid range 230–320) of the 480 aa protein sequence. To explore the potential functional significance of these amino acid differences, the relationship of *A. baumannii* Wzi to the established tertiary structure of Wzi from *E. coli* O9a:K30 was examined.

**Table 3:** Percentage amino acid sequence identity of *wzi* types.

Type^a^	Type 4	Type 1	Type 2	Type 3
Type 4	100	89.19	88.12	88.54
Type 1	89.19	100	93.54	90.83
Type 2	88.12	93.54	100	93.75
Type 3	88.54	90.83	93.75	100

^a^Type 1 = AB5075-UW (ABUW_2898 in CP008706.1), type 2 = ATCC17978 (AUO97_12210 in CP018664.1), type 3 = ACICU (DMO12_03033 in CP031380.1), type 4 = LAC4 (BBX32_12830 in CP018677.1).

**Figure 7:**
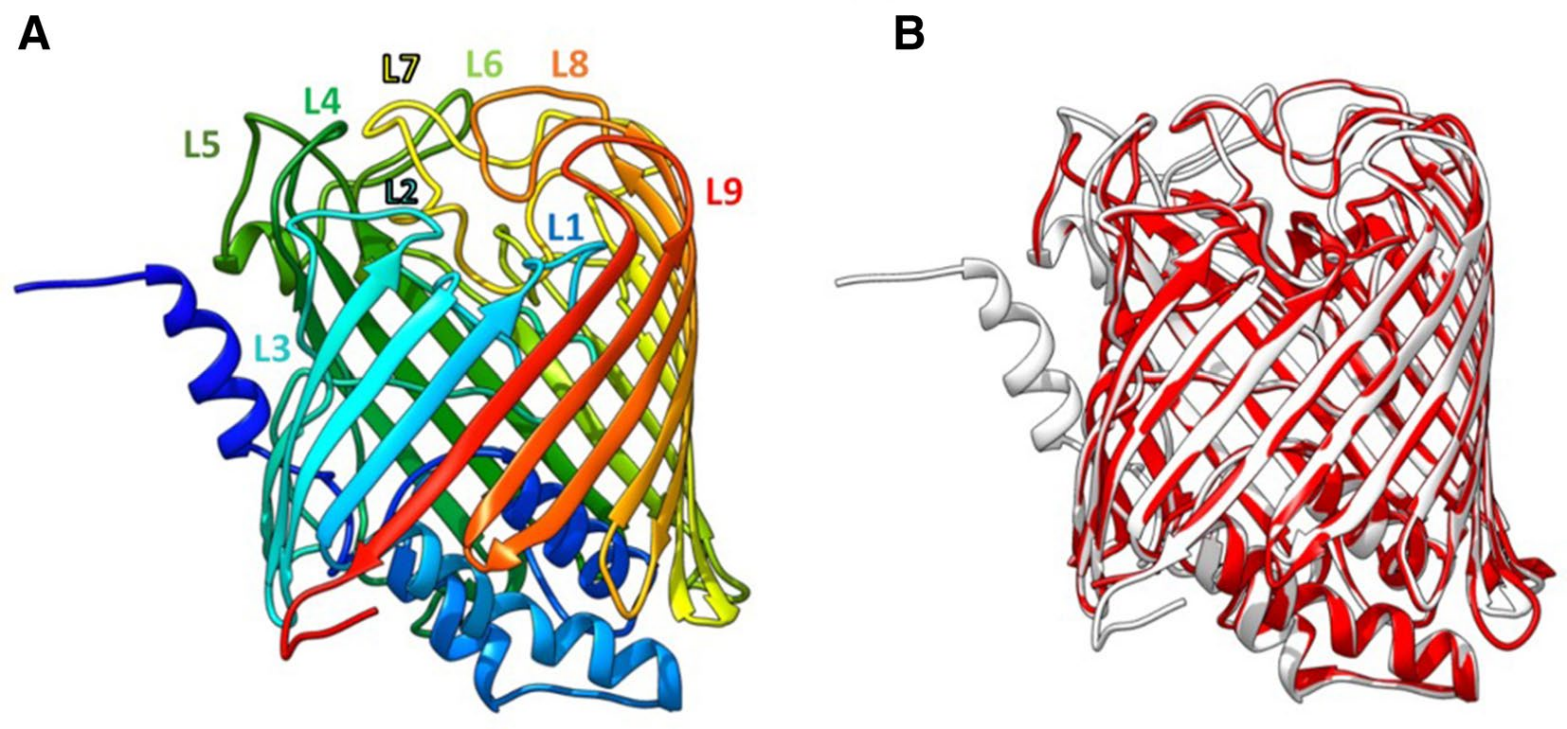
(**A**) Predicted tertiary structure of Wzi type 1 encoded by *A. baumannii* AB5075-UW showing the location of nine extracellular loops (L1-L9). (**B**) *Acinetobacter baumannii* AB5075-UW Wzi structure (white) overlaid on *E. coli* K30 Wzi reference structure (red; PDB: c2ynkA and Bushell et al. 2013).

The tertiary structure of the AB5075-UW Wzi (ABUW_2898; Type 1) was modeled using Phyre^2^ software, which revealed a match of 100% confidence with 48% identity (94% coverage: residues 23–476) to the *E. coli* O9a:K30 Wzi structure (PDB: c2ynkA)^15^. The predicted type 1 Wzi three-dimensional structure (Fig. 7A) consists of an 18-stranded β-barrel fold that includes a helical periplasmic bundle and an arrangement of nine loops on the extracellular side. This is consistent with the *E. coli* O9a:K30 Wzi structure, and an overlay of the two demonstrates a close match (Fig. 7B).

Previously, an interaction between the *E. coli* K30 CPS structure and the extracellular loops of Wzi was demonstrated, with L3, L6 and L7 loops found to be critical for CPS assembly on the cell surface^15^. Therefore, the extracellular loops were identified in the modeled tertiary structure of the AB5075-UW Wzi (numbered L1-L9 in Fig. 7A), and the amino acid sequences of the loops were identified in the multiple sequence alignment of the four *A. baumannii* Wzi types (Supplementary Fig. S2). The predicted L3, L6 and L7 were found to be mostly conserved, whereas the majority of amino acid sequence differences between all types (range 230–320) were identified in the sequence of L5. Further work will be needed to directly assess the importance of the extracellular loops in Wzi function in *A. baumannii*.

## Discussion

The *A. baumannii* CPS affords a remarkable amount of cell-surface diversity between different isolates with more than 128 distinct structural forms predicted^14^. The intrinsic capacity of the organism to frequently exchange and replace CPS biosynthesis genes^24^ indicates that there are likely many more CPS forms yet to be discovered. This extreme heterogeneity complicates therapeutic strategies that target specific CPS structures. However, damaging or completely removing this surface barrier could present a viable alternative approach, yet research on the precise functions of *A. baumannii* CPS biosynthesis proteins is still developing.

In this study, we confirm that Wzi is required for the proper assembly of a tight CPS layer on the cell surface, with the deletion of *wzi* resulting in a reduction of cell-associated CPS with a reciprocal increase of CPS in the extracellular surrounds. Though a small amount of CPS material (~ 20%) is still observed on the cell surface in *wzi* mutants, it may be CPS actively passing through the outer membrane completing its export to the cell surface. Nonetheless, the integrity of the CPS barrier as a discrete layer surrounding the cell is compromised.

Analysis of the AB5075-UW wildtype showed a small amount of cell-free CPS. In a previous study, both cell-bound and cell-free CPS material has been detected for another wildtype isolate^10^, suggesting that CPS shedding may be a natural phenomenon in the species regulated by Wzi expression. Thus, it is possible that regulation of CPS retention on the cell surface may be necessary for different environmental contexts and stresses or during different stages of host infection. Further work will be needed to assess the expression of Wzi while under stress, and to confirm the promoter region and any potential regulators.

The importance of proper assembly of CPS on the cell surface for biofilm formation was also assessed as previous studies have shown that the *itr* initiating transferase is critical for the development of uniform biofilm structures^8^. Interestingly, the retention of CPS on the cell surface was shown to have little importance for biofilm, though a marked reduction was observed for the *itrA1* mutant as expected^8,20^, indicating that CPS presence rather than cell-surface retention is important. However, in *A. baumannii*, K-unit oligosaccharides that make up the CPS are also used for *O*-glycosylation of proteins^8^, therefore loss of *O*-glycosylation via an *itr* deletion may account for biofilm defects. Though a role for Wzi in biofilm formation was not established, a previous study has shown that an AB5075 *wzi* (ABUW_2898) mutant displays decreased resistance to normal human serum^25^. This suggests that the proper assembly and retention of the CPS on the cell surface is likely to play a more significant role in the evasion of human immune defenses.

The chromosomal K locus is a recombination hotspot^24^, and while nearly all *wzi* genes are located away from this genomic region in *A. baumannii* (Fig. 1A), sequence diversity in *wzi* has so far gone undetected. The amount of sequence variation at the *wzi* locus was a surprising finding, exceeding that observed for the intrinsic *oxaAB* gene with > 95% nucleotide sequence identities identified across all genomes (Supplementary Table S1). However, as five different *wzi* types were identified amongst > 9300 *A. baumannii* genomes, it is possible that multiple imports of the *wzi* gene into the species have occurred. The results in this study indicate that variation at the *wzi* and K locus is not concomitant, suggesting that the two regions are evolving independently. However, a significant association between the *wzi* type and the first sugar of the CPS structure was observed. This raises the possibility that acquisition, and successful maintenance of K locus types is restricted by the *itr* initiating transferase gene due to the possible dependence on the *wzi* gene type to produce a tight CPS layer on the cell surface. Further studies will be needed to confirm if Wzi types have stringent substrate specificities.

## Materials and methods

### Bacterial strains and cultivation

*Acinetobacter baumannii* AB5075-UW (K25 capsule type^26^) and derivative T26 insertion mutants (Table 1) were obtained from the *A. baumannii* AB5075 transposon mutant library^27^. Bacteria were routinely grown in Luria–Bertani (LB) media at 37 °C with or without shaking at 200 rpm, and complemented strains were selected used 50 ug/ml apramycin. Optical density (OD) was measured by absorbance at 600 nm using a densitometer.

### Whole genome sequencing and analysis

Whole genome sequences were obtained by Illumina NextSeq 500 using the Nextera-XT Library at the Forensic and Scientific Services Laboratory (QLD Health, Brisbane). Paired-end short read data were de novo assembled into contigs using the SPAdes algorithm optimized via the Unicycler v 0.4.8 assembly pipeline^28^. The locations of transposon insertions were identified using Clustal Omega^29^. Snippy v 3.2 (https://github.com/tseemann/snippy) was used to determine the number of single nucleotide polymorphisms (SNPs) between mutant sequences and the complete reference genome sequence of wild type AB5075-UW (GenBank accession number CP008706).

### Complementation of *A. baumannii* wzi::T26 mutants

Complementation of *wzi::T26* mutants was performed using an overlap extension PCR protocol to generate a chimeric cassette (6.5 kb) targeted for insertion into the *att*Tn*7* site in the AB5075 chromosome^30^. Briefly, primary PCR reactions using oligonucleotides (listed in Supplementary Table S5) consisting of additional sequence homologous to the intended adjacent amplicon were used to generate individual cassette fragments (Fig. 1C). These fragments included 2 kb of sequence identical to either side of the *att*Tn*7* insertion site, the predicted *wzi* promoter region and *wzi* gene from AB5075 (GenBank accession number CP008706.1, base range 2,905,472 to 2,906,917) and an *aacC4* gene (GenBank accession number KM670336.1, base range 139,244–140,020) conferring resistance to apramycin. Amplicons were assembled into a single cassette using a secondary PCR reaction with the outermost oligonucleotides. PCR was performed using a high-fidelity DNA polymerase (Phusion, Thermo Fisher), and the assembled cassette was Sanger sequenced for confirmation.

Complementation was performed using natural transformation of the chimeric cassette into *wzi*-deficient transposon mutants (*wzi*::T26-1, *wzi*::T26-2, and *wzi*::T26-3). LB media was inoculated with a single colony of a mutant strain then incubated at 37 °C until OD_600nm_ 1.0 was achieved, then cultures were diluted 1:100 in Tryptone medium (5 g/L). Equal volumes of diluted culture and cassette DNA (200 ng/ml) were mixed and then spotted on Tryptone medium containing 2% low electroendosmosis agar in a microcentrifuge tube. Following overnight incubation at 37 °C, cells were plated onto LB agar containing 50 mg/ml apramycin and resulting colonies were screened by PCR to identify transformants. Successful transformants were confirmed by PCR across insertion sites and Sanger sequencing.

### CPS extraction and SDS-PAGE visualization

Prior to extraction, overnight cultures at equivalent OD_600nm_ were centrifuged to separate cells from the supernatant. CPS attached to the cell surface was purified from whole-cell lysates (cell fractions) using the phenol:chloroform extraction method described previously^17,31^. CPS shed from the cell surface was extracted from the supernatant fractions of the same cultures by adding 4 × volume of ice-cold ethanol and precipitating at − 20 °C overnight. Samples were then centrifuged 12,000 rpm at 4 °C for 15 min, and pellets were resuspended in sterile milli-Q water. Equivalent volumes of purified CPS samples were subjected to SDS-PAGE (4% stacking and 16% separating tricine gels), and gels were stained overnight in Alcian blue buffer (25% isopropanol, 7% acetic acid solution and 0.05% w/v Alcian blue) as described previously^32^. Gels were imaged using a ChemiDoc XRS gel imaging system. Densitometric analysis was performed using ImageJ software^33^.

### Transmission electron microscopy

Mid-exponential phase subcultures were centrifuged at 1000 rpm for 30 min. Cell pellets were stained for 10 min with 2.5% glutaraldehyde, 75 mM lysine, and 0.075% ruthenium red in 0.1 M sodium cacodylate buffer, then briefly centrifuged at 1000 rpm for 5 min. Cell pellets were then washed twice gently with 2.5% glutaraldehyde in 0.1 M sodium cacodylate buffer, then centrifuged at 1000 rpm for 5 min before incubating in 2.5% glutaraldehyde and 0.1 M sodium cacodylate buffer for 1 h. Samples were then treated with 0.1 M sodium cacodylate buffer for 15 min prior to centrifugation at 1000 rpm for 5 min, and this process was repeated four times. The Central Analytical Research Facility (CARF) at the Queensland University of Technology (QUT) prepared sections cut 50 nm thin that were mounted on 75 mesh copper grids, and post-stained for 5 min with 2% uranyl oxalate and 3 min with lead citrate. Sections were imaged on a JEOL 1400 transmission electron microscope model F216 (TVIPS GmBH) at 100 kV, spot size 2.

### Biofilm assay

Biofilm growth assays were performed in a Calgary biofilm device (CBD) (MBEC assay; Innovotech Inc., Canada). Overnight bacterial cultures in LB were diluted to 10^6^ CFU/ml in LB and used to inoculate the plate with 130 μL of culture per well. The CBD was incubated for 24 h with shaking (150 rpm) at 37 °C in 95% relative humidity. Following 24 h of growth, biofilms were washed once in PBS to remove non-adherent cells and then sonicated for 20 min at 20 °C. At least 2 representative wells per strain per experiment were chosen at random and then serially diluted and spotted onto LB agar plates in duplicate to determine viable CFU recovered from each peg biofilm. Plates were incubated overnight at 37 °C with colonies counted the following day to obtain log10(CFU/mL) values for each strain. Group means were compared by one-way ANOVA with Tukey’s multiple comparisons post hoc test.

### Bioinformatics analysis

A total of 9459 genome assemblies listed under taxonomy *Acinetobacter baumannii* were downloaded from NCBI (https://www.ncbi.nlm.nih.gov/assembly/) on the 26th of August, 2021. The presence of the intrinsic *oxaAB* gene was used to confirm the species as *baumannii* as performed previously^14^, and *oxaAB-*negative genomes were removed from further analyses. Genomes were initially screened for *wzi* type 1 from AB5075-UW (locus tag ABUW_2898 in GenBank accession number CP008706.1). Matches with < 100% coverage and/or < 80% identity were then assessed for sequence quality using QUAST (http://bioinf.spbau.ru/quast). Poor quality genomes, defined using parameter described previously in Wyres et al. 2020 (> 300 contigs and/or total length of genome < 3.6 Mb), and genomes where *wzi* was either found broken across more than one contig or < 100% coverage to *wzi* type 1, were also removed. Therefore, a total of 9342 genome assemblies (listed in Supplementary Table S1) were included in further analyses.

Representative *wzi* nucleotide sequences were extracted from 109 genome assemblies (see Supplementary Table S2 for accession numbers and traits). K locus types were assigned using command-line *Kaptive* v 0.7 (https://github.com/katholt/Kaptive) with the *A. baumannii* KL reference database and default parameters^14^. Sequence types were assigned using the *A. baumannii* Pasteur Multi-locus sequence typing (MLST) scheme available at https://pubmlst.org/bigsdb?db=pubmlst_abaumannii_pasteur_seqdef.

A multiple pairwise *wzi* nucleotide sequence alignment was constructed using MUSCLE (https://www.ebi.ac.uk/Tools/msa/muscle/), and used to infer a maximum likelihood tree based on 1000 replicates using RAxML v 8.0.0 with the GTRGAMMA model^34^. The resulting tree was visualized, mid-rooted, and annotated using the iTOL web-interface^35^ and edited using Adobe Illustrator. Stacked bar and mosaic plots were created in RStudio v 1.2.5033^36^ using ggplot^37^ and vcd^38^ packages.

Percentage identity matrices and multiple pairwise alignments to visualise nucleotide and protein sequences were constructed using CLUSTAL Omega (https://www.ebi.ac.uk/Tools/msa/clustalo/). Phyre^2,39^ was used to predict the tertiary structure of the Wzi protein from AB5075-UW via sequence threading to the *E. coli* K30 Wzi reference (PDB: 2YNK). The predicted structure was visualized using Chimera software^40^.

## Supplementary Information

Supplementary Information 1.

Supplementary Information 2.

## Data Availability

The dataset analysed during the current study are 9459 *Acinetobacter baumannii* genome assemblies available in the NCBI repository (https://www.ncbi.nlm.nih.gov/assembly/?term=acinetobacter+baumannii) as of the 26th August 2021.
