# Supplementary material for: The Wzi outer membrane protein mediates assembly of a tight capsular polysaccharide layer on the Acinetobacter baumannii cell surface
Source: Sci Rep. 2021 Nov 5;11:21741. doi: 10.1038/s41598-021-01206-5 (PMC8571296; doi:10.1038/s41598-021-01206-5)
Supplement: Supplementary file 1 — Supplementary Information 1. [file 41598_2021_1206_MOESM1_ESM.pdf]

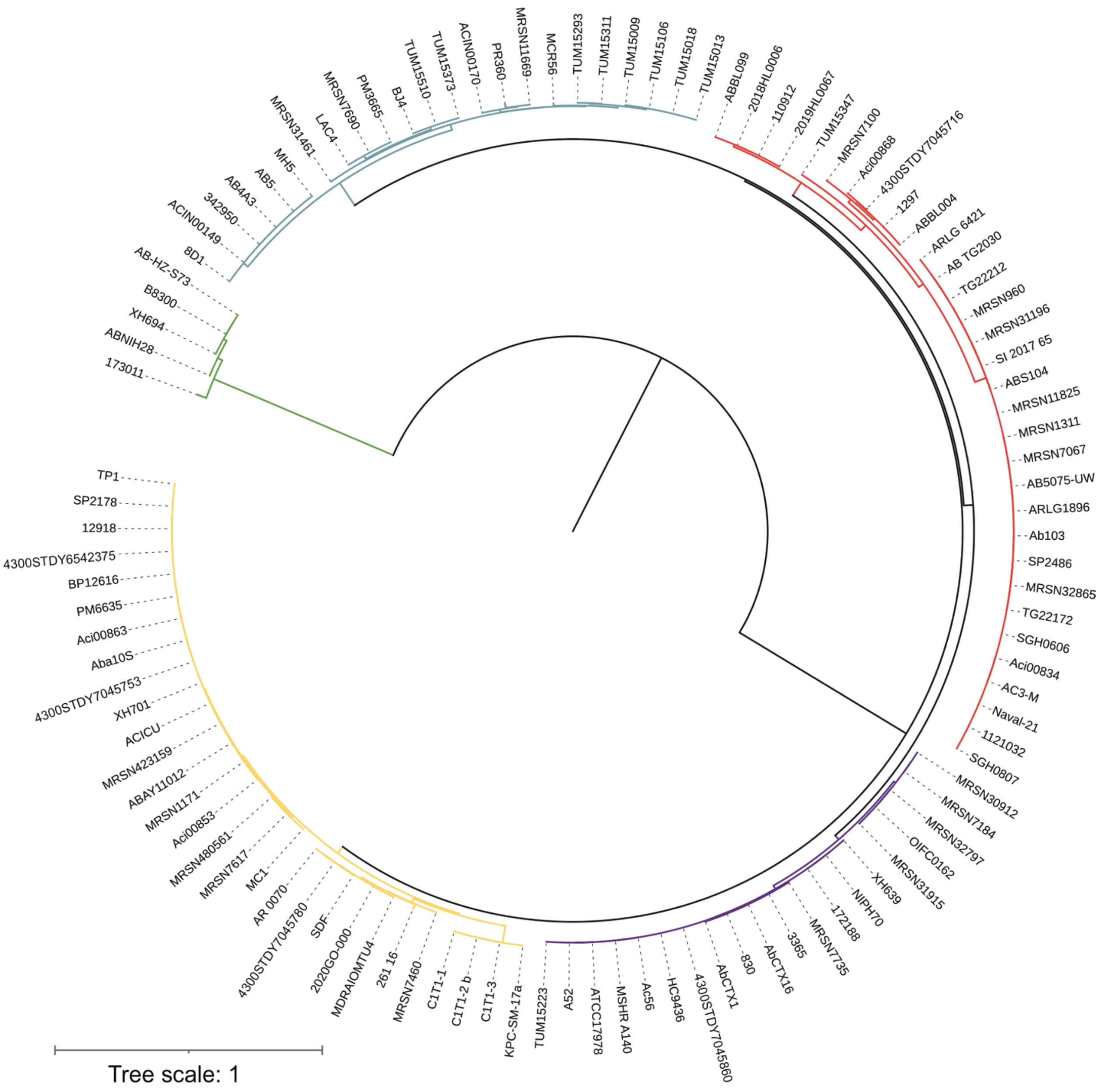

Figure S1. Maximum likelihood tree of *wzi* nucleotide sequences from 109 A. baumannii genome assemblies. Tree is based on 1000 replicate trees constructred using RAxML v 8.0.0 with the GTRGAMMA model and visualised in iTOL. Bootstrap values are not shown. Tree scale is below. Major phylogeny clades indicating *wzi* types are coloured differently. Assembly accession numbers are listed in Supplementary Table S1.

```
Type4      MFLRKTLSIALLATASSAVFAQGLVLNNDDLRTDLNWLNQQGVINISTSTWPLSGDEIQR   60
Type1      MFLRKTLSIALLATASSAVFAQGLVLNNDDLRTDLNWLNQQGVISISTSTWPLSGDEIQR   60
Type2      MFLRKTLSIALLATASSAVFAQGLVLNNDDLRTDLNWLNQQGVINISTSTWPLSGDEIQR   60
Type3      MFLRKTLSIALLATASSAVFAQGLVLNNDDLRTDLNWLNQQGVINISTSTWPLSGDEIQR   60
           ********************************************.***************

                                               Loop 1
Type4      ALSQAKVTHPAQQKVINSVLNALKADNDTVKVGAFAETDIKNIPQAFGDNQKSQYQGSLE   120
Type1      ALSQAKVTHPAQQKVINSVLNALKADNNTVKVGAFAETDIKNIPQAFGDNQKAQYQGSLE   120
Type2      ALSQAKVTHPAQQKVINSVLNALKADNNTVKVGAFAESDIKNIPQAFGDNQKSQYQGSLE   120
Type3      ALSQAKVTHPAQQKVINSVLNALKADNDTVKVGAFAETDIKNIPQAFGDNQKSQYQGSLE   120
           ***************************:*********:**************:*******

                             Loop 2                        Loop 3
Type4      FNAGGENWDAKIRVNAEKDPQIDSGHDVNVEGSYVAGKLWNQWLVAGQIPTWWGPGHDGS   180
Type1      FNAGGENWDAKIRVNAEKDPQIDSGHDVNVEGSYVAGKLWNQWIVAGQIPTWWGPGHDGS   180
Type2      FNAGGENWDAKIRVNAEKDPQIDSGHDVNVEGSYVAGKLWNQWIVAGQIPTWWGPGHDGS   180
Type3      FNAGGENWDAKIRVNAEKDPQIDSGHDVNVEGSYVAGKLWNQWLVAGQIPTWWGPGHDGS   180
           *******************************************:****************

                                                 Loop 4
Type4      LIRGDASRPVYGVTMQRAVQDAFENKWLSWIGPWQYQLFAGQLDDYKAVPHAKLLGMRVT   240
Type1      LIRGDASRPVYGVTAQRAVQNAFETKWLSWIGPWQYQAFAGQLDDYKAVPDAKLIGLRVT   240
Type2      LIRGDASRPVYGVTAQRAVQNAFETKWLSWIGPWQYQAFAGQLDDYKAVPDAKLIGLRLT   240
Type3      LIRGDASRPVYGVTAQRAVQNAFETKWLSWIGPWQYQAFAGQLDDYKAVPHAKLLGLRLT   240
           ************** *****:***.************ ************.***:*:*:*

                             Loop 5
Type4      ARPVPALEIGASRAIQIGGDGQPDSFKAYWNAVIGKDNGCTENSCVGEDNASNQLAGFDA   300
Type1      AQPLPYIELGASRAIQWGGDGRPESLSSLWDAFVGNK----DNGGTGEPDPSNQIAGFDG   296
Type2      AQPLPYLELGASRTIQWGGDGRSESFSSLWDAIKGND----NVYGD-TENPSNQLAGFDG   295
Type3      ARPLPYLELGASRTLQWGGEGRSESWDSLWNAIKGND----NVYDS-DEDRSNQIAGFDA   295
           *:*:* :*:****::* **:*: :* .: *:*. *:.    :       : ***:****.
                                Loop 5
Type4      RLQLQPLFNIPVSVYGQYIGEDEAGGLPSKKMYLAGADYSSMINNMPYQIYAEWADTRTN   360
Type1      RLLLQPLFQVPVSLYGQYVGEDEAGGLPSKKMYLAGVDYSSSYNNMPYQLYAEWADTRTN   356
Type2      RLLLQPLLNIPVSLYGQYVGEDEAGYLPSKKMYLAGVDYSSSYNDMPYQLYAEWADTRTN   355
Type3      RLNLQSLINAPVGIYGQYVGEDEAGLLPSKKMYLAGVDYSSSYNNMPYQLYAEWADTRTN   355
           ** ** *:: **.:****:****** **********.****  *:****:**********
           Loop 7
Type4      GDIRGISYNHHQYTDGYYQHGYPLGHAMGGDGQMYSVGGDIRFDVMNRLSGRAMVVKVNQ   420
Type1      GDVRGISYAHSTYKDGYYQHGYPLGHAMGGDGQMYSVGGDIRFDVMNRLSGRAMVVKVNQ   416
Type2      GDVKSISYTHSVYKDGYYQHGFPLGHAMGGDGQMYSVGGDIRFDVMNRLSGRAMVVKVNQ   415
Type3      NDVKGISYNHYVYKDGYYQHGFPLGHAMGGDGQMYSVGGDIRFDVMNRLSGRAMVVKVNQ   415
           .*::.*** *  *.*******:**************************************
           Loop 8                                 Loop 9
Type4      SNLAINKAFPKDYEIKALDLTWTHFIKPDLPLKINGWVSDSDLEGNDAGASIGIEIPLER   480
Type1      SNLAINKAFPKDDEIKALDLTWTHFIKPDLPLKINGWVSDSDLEGNDAGASIGIEIPLER   476
Type2      SNLAINKAFPKDDEIKALDLTWTHYIKPDLPLKINGWVSDSDLEGNDAGASIGVEIPLER   475
Type3      SNLAINKAFPKDDEIKALDLTWTHYIKPDLPLKINGWVSDSDLEGNDAGASIGVEIPLER   475
           ************ ***********:****************************:******

Type4      KMFGF   485
Type1      KMFGF   481
Type2      KMFGF   480
[illegible]
```

Figure S2. Multiple sequence alignment of the four main Wzi sequence types. Alignment constructed by CLUSTAL Omega (https://www.ebi.ac.uk/Tools/msa/clustalo/). Representative types are: Type 1 = AB5075-UW (ABUW_2898 in CP008706.1), type 2 = ATCC17978 (AUO97_12210 in CP018664.1), type 3 = ACICU (DMO12_03033 in CP031380.1), type 4 = LAC4 (BBX32_12830 in CP018677.1). Bold face indicates loops identified by structural comparison to *E. coli* K30 Wzi in Fig. 7, with red indicating loops identified in *E. coli* that may interact with the CPS.

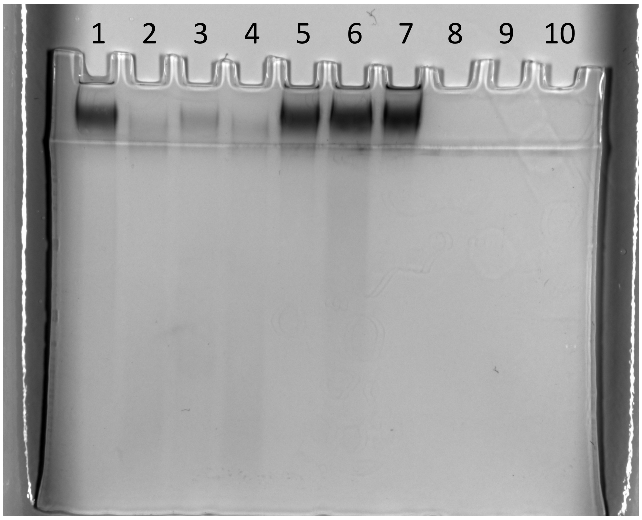

Gel 1	
**Lane**	**Sample**
1	AB5075 WT, attached CPS, 6 OD equiv.
2	*wzi*::T26-1, attached CPS, 6 OD equiv.
3	*wzi*::T26-2, attached CPS, 6 OD equiv.
4	*wzi*::T26-3, attached CPS, 6 OD equiv.
5	*wzi*::T26 -1 attTn7-*wzi*, attached CPS, 6 OD equiv.
6	*wzi*::T26 -2 attTn7-*wzi*, attached CPS, 6 OD equiv.
7	*wzi*::T26 -3 attTn7-*wzi*, attached CPS, 6 OD equiv.
8	Blank
9	Blank
10	Blank

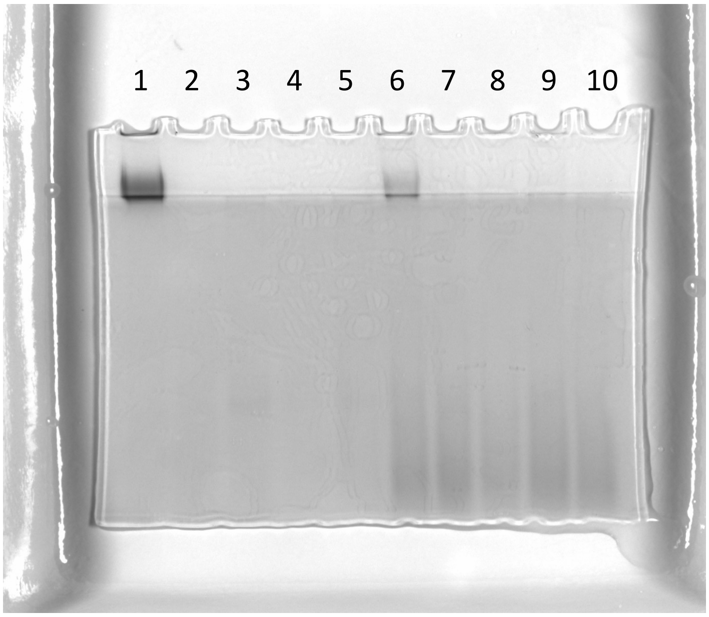

Gel 2	
**Lane**	**Sample**
1	AB5075 WT, attached CPS, 6 OD equiv.
2	*Other sample*
3	*itrA1*::T26, attached CPS, 6 OD equiv.
4	*Other sample*
5	*Other sample*
6	AB5075 WT, shed CPS, 6 OD equiv.
7	*Other sample*
8	*itrA1*::T26, shed CPS, 6 OD equiv.
9	*Other sample*
10	*Other sample*

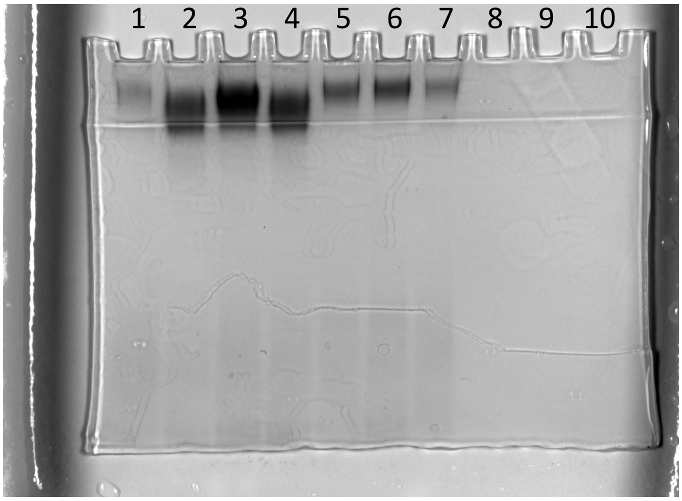

Gel 3	
**Lane**	**Sample**
1	AB5075 WT, shed CPS, 6 OD equiv.
2	*wzi*::T26-1, shed CPS, 6 OD equiv.
3	*wzi*::T26-2, shed CPS, 6 OD equiv.
4	*wzi*::T26-3, shed CPS, 6 OD equiv.
5	*wzi*::T26 -1 attTn7-*wzi*, shed CPS, 6 OD equiv.
6	*wzi*::T26 -2 attTn7-*wzi*, shed CPS, 6 OD equiv.
7	*wzi*::T26 -3 attTn7-*wzi*, shed CPS, 6 OD equiv.
8	Blank
9	Blank
10	Blank

Figure S3. Original uncropped SDS-PAGE gels shown in Fig. 2. Samples for each lane are indicated in the adjacent tables. Yellow highlighted lanes are sample shown in Fig. 2.
